# Association of Adiponectin, Leptin and Resistin Plasma Concentrations with Echocardiographic Parameters in Patients with Coronary Artery Disease

**DOI:** 10.3390/diagnostics11101774

**Published:** 2021-09-26

**Authors:** Kamila Puchałowicz, Karolina Kłoda, Violetta Dziedziejko, Monika Rać, Andrzej Wojtarowicz, Dariusz Chlubek, Krzysztof Safranow

**Affiliations:** 1 Department of Biochemistry and Medical Chemistry, Pomeranian Medical University, 70111 Szczecin, Poland; viola@pum.edu.pl (V.D.); carmon12@gmail.com (M.R.); dchlubek@pum.edu.pl (D.C.); chrissaf@mp.pl (K.S.); 2 Medfit Karolina Kłoda, 70240 Szczecin, Poland; wikarla@gazeta.pl; 3 Department of Cardiology, Pomeranian Medical University, 70111 Szczecin, Poland; wojtaro@pum.edu.pl

**Keywords:** adipose tissue, adiponectin, cardiac diastolic function, cardiac remodelling, cardiac systolic function, echocardiography, heart failure, leptin, myocardial infarction, resistin

## Abstract

The imbalanced network of adipokines may contribute to the development of systemic low-grade inflammation, metabolic diseases and coronary artery disease (CAD). In the last decade, three classic adipokines—adiponectin, leptin and resistin—have been of particular interest in studies of patients with CAD due to their numerous properties in relation to the cardiovascular system. This has directed our attention to the association of adipokines with cardiac structure and function and the development of heart failure (HF), a common end effect of CAD. Thus, the purpose of this study was to analyse the associations of plasma concentrations of adiponectin, leptin and resistin with parameters assessed in the echocardiographic examinations of CAD patients. The presented study enrolled 167 Caucasian patients (133 male; 34 female) with CAD. Anthropometric, echocardiographic and basic biochemical measurements, together with plasma concentrations of adiponectin, leptin and resistin assays, were performed in each patient. Adiponectin concentrations were negatively associated with left ventricular ejection fraction (LVEF) and shortening fraction (LVSF), and positively associated with mitral valve E/A ratio (E/A), left ventricular end-diastolic volume (LVEDV), left ventricular end-diastolic diameter (LVEDD), left ventricular end-systolic diameter LVESD, and left atrium diameter (LAD). Resistin concentrations were negatively associated with E/A. Leptin concentrations, although correlated with HF severity assessed by the New York Heart Association (NYHA) Functional Classification, were not independently associated with the echocardiographic parameters of cardiac structure or function. In conclusion, adiponectin and resistin, but not leptin, are associated with the echocardiographic parameters of cardiac remodelling and dysfunction. These associations suggest that adiponectin and resistin might be involved in mechanisms of cardiac remodelling or compensative response. We also suggest the possible benefits of adiponectin and resistin level measurements in the monitoring of patients with CAD.

## 1. Introduction

Coronary artery disease (CAD) is the leading global problem of public health because it is related to high morbidity and mortality and is a large burden for financial and health systems [1]. Both cardiovascular diseases (CVD) and cardiovascular risk factors, such as hypertension (HT), metabolic syndrome, dyslipidaemia and type 2 diabetes mellitus (T2DM), are closely related to obesity [2,3]. A breakthrough in understanding the relationship between obesity and CAD was the change in perception of adipose tissue functions. Nowadays, adipose tissue is understood not only for its role in energy storage, thermal and mechanical isolation, but also as a dynamic endocrine organ [4,5]. The role of adipose tissue mediators, both in health and disease, is played by a growing family of proteins with pleiotropic functions called adipokines [5].

The consequence of chronic positive caloric balance is adiposopathy, which is a pathologic state of adipose tissue characterised by anatomic and functional disturbances with adverse endocrine and immune responses [6]. Changes in body fat distribution and an imbalanced network of adipokines may contribute to development of systemic low-grade inflammation, metabolic diseases and CAD [5]. The anatomical depot of adipose tissue is a factor that significantly affects the profile of secreted adipokines. In obesity, the mass of visceral adipose tissue (VAT) and epicardial adipose tissue (EpAT) is significantly increased. Both VAT and EpAT promote a pro-inflammatory secretome profile, which modulates arterial vessel and heart functions. Increased volumes of these adipose tissue depots are associated with CAD development [4,7]. Research regarding CAD has focussed on three classic adipokines—adiponectin, leptin and resistin—which have been shown to be associated with the development of CAD [8,9,10]. It is believed that circulating adipokines may serve as potential predictive and prognostic markers in patients with CVD [8,9,10,11,12,13,14,15]. This has prompted us to take a closer look at the association of adipokines with cardiac structure and function and the development of heart failure (HF), a common end effect of CAD.

Adiponectin is a multifunctional adipokine released almost exclusively by adipocytes with a broad range of target tissues, including adipose tissue, skeletal muscles, liver and cardiovascular system. This adipokine is considered to be a protective mediator in CAD due to its numerous beneficial properties, among them anti-insulin resistance, anti-inflammatory, anti-oxidant, anti-apoptotic, vasodilatant and cardioprotective effects [4,16,17]. A characteristic feature of patients with obesity (especially with abdominal fat accumulation), insulin resistance, T2DM and CAD is hypoadiponectinaemia [18,19], which occurs in response to the low-grade inflammation accompanying these disorders [20]. Hypoadiponectinaemia is associated with an increased incidence of metabolic syndrome [21] and T2DM [22], and increased carotid intima-media thickness [23]. However, there is no clear position on the association of low adiponectin level with risk of CVD [24,25,26,27,28,29]. Adiponectin concentrations are markedly increased in advanced CVD states such as HF [11,12,13] by circulating brain natriuretic peptide (BNP) levels [20]. Despite the beneficial effects of adiponectin, high circulating adiponectin concentrations do not necessarily appear to translate into better clinical outcomes in patients with CVD [30].

Leptin, like adiponectin, is mainly secreted by adipocytes. The main function of leptin is suppression of the appetite and food intake signal in the hypothalamus, in addition to regulating energy expenditure [10]. It is a controversial issue among researchers whether leptin protects or damages the cardiovascular system. On one hand, studies conducted in leptin-deficient or leptin-receptor-deficient rodents indicated beneficial effects of leptin on tissue insulin response, lipid profile, body weight and cardiac metabolism (keeping the balance between glucose metabolism and fatty acid oxidation). Moreover, leptin has been suggested to protect the heart from lipotoxicity under cardiac stress conditions [31]. On the other hand, high concentrations of leptin are characteristic of conditions such as obesity, T2DM [31] and CAD [32] and are not associated with beneficial effects due to the development of leptin resistance [33]. The presence of metabolic syndrome appears to significantly modulate the beneficial effects associated with leptin [34]. Hyperleptinaemia is associated with the development of HT and CVD. Leptin is considered an important component of vascular and heart dysfunction pathogenesis in obese people due to the promotion of low-grade systemic inflammation, impaired cardiac metabolism and vasoactive, pro-thrombotic and pro-fibrotic activity [10,31].

Human resistin is mainly secreted by peripheral blood mononuclear cells and resident macrophages. Among the wide range of effects of resistin, the following should be mentioned: the stimulation of inflammation, disruption of insulin-signalling in myocytes and hepatocytes, induction of vascular endothelium dysfunction, stimulation of very low-density lipoprotein (VLDL) production and suppression of low-density lipoprotein (LDL) receptors [5,9]. Studies in animal models have indicated that resistin may directly affect the myocardium, promoting cardiac hypertrophy and dysfunction [35,36,37]. In this way, resistin participates in obesity-related diseases, such as dyslipidaemia, atherosclerosis and CVD.

Due to the growing need to fully understand the impact of cytokines secreted by adipose tissue on cardiac structure and function, the aim of this study was to analyse the associations between adiponectin, leptin and resistin plasma concentrations, and the parameters assessed in the echocardiographic examinations of CAD patients.

## 2. Materials and Methods

### 2.1. Study Design and Participants

This study enrolled 167 patients (133 male; 34 female) with CAD aged 59.2 ± 8.4 years. This Caucasian, Polish population was treated at the Clinical Department of Cardiology of the Pomeranian Medical University in Szczecin. This same study group was described in our previous research [38,39]. The inclusion criteria were: diagnosis of CAD based on the presence of at least one coronary lesion on coronary angiography (≥40% diameter stenosis of the left main coronary artery, ≥50% stenosis of one of the three major epicardial arteries or ≥70% stenosis of a branch). All patients received optimal pharmacological treatment and were clinically stable. Neither acute coronary syndromes nor HF exacerbations were present, nor were any revascularisation procedures conducted within one month before enrolment in the study. Further exclusion criteria included: haemodynamically significant congenital or acquired valve diseases, advanced renal failure (serum creatinine > 2.5 mg/dL), malignant neoplasms, rheumatoid arthritis or other autoimmune connective tissue diseases.

HF was confirmed in 70 (42%) patients based on clinical symptoms, echocardiography and BNP plasma concentration. The extent of HF was evaluated with the New York Heart Association (NYHA) Functional Classification and the severity of CAD symptoms was evaluated by the Canadian Cardiovascular Society (CCS) Angina Grading Scale. Basic anthropometric parameters, systolic and diastolic blood pressures (SBP and DBP) and heart rate (HR) were measured for each patient. The body mass index (BMI) and waist-to-hip ratio (WHR) were also calculated.

Study protocol conformed to the Declaration of Helsinki principles and was approved by the Pomeranian Medical University in Szczecin Ethics Committee (No. BN-001/124/03). Informed consent was obtained from each patient. The clinical characteristics, and laboratory and echocardiographic parameters of the study group are shown in Table 1. Abbreviations for these parameters used throughout the manuscript are also presented in the table.

### 2.2. Blood Samples and Biochemical Measurements

Fasting blood samples were collected into tubes with EDTA for plasma and clotting activator for serum separation. The concentrations of blood haemoglobin, fasting plasma glucose, serum lipid profile (total, high-density lipoprotein (HDL) and LDL cholesterol levels and triacylglycerols), creatinine and uric acid were measured routinely in a hospital laboratory. Plasma BNP concentrations were measured with the AxSym assay (Abbott Laboratories, Abbott Park, IL, USA). Estimated glomerular filtration rate (eGFR) was calculated using the simplified Modification of Diet in Renal Disease Study (MDRD) equation. Plasma samples for the measurements of adipokines and BNP were stored at −80 °C until analysed.

### 2.3. Measurements of Adipokine Concentrations

Concentrations of adiponectin, leptin and resistin in plasma were assessed with commercially available ELISA kits (Quantikinine R&D Systems Inc., Abingdon, UK; catalogue numbers DRP300, DLP00 and DRSN00, respectively) according to the manufacturer’s protocol. Absorbance was read at 450 nm (with correction at λ = 540 nm) using automated Microplate Reader EnVision 2104 (Perkin Elmer, Waltham, MA, USA).

### 2.4. Echocardiography

Echocardiographic assessment of the cardiac anatomy and function was performed in each patient by one experienced cardiologist (A.W.) using Vivid 7 Pro (GE Healthcare, Boston, MA, USA) equipment. The cardiac parameters were acquired and interpreted according to the recommendations of the American Society of Echocardiography [40]. The following echocardiographic parameters were recorded: left ventricular mass index (LVMI), left ventricular end-diastolic volume (LVEDV), left ventricular end-diastolic diameter (LVEDD), left ventricular end-systolic diameter (LVESD), intraventricular septal end-diastolic thickness (IVSd), posterior wall end-diastolic thickness (PWd), ascending aorta diameter (Ao), left atrium diameter (LAD), right ventricular end-diastolic diameter (RVEDD), right ventricular systolic pressure (RVSP), left ventricular ejection fraction (LVEF), left ventricular shortening fraction (LVSF), mitral valve E/A ratio (E/A), mitral valve deceleration time (DT), isovolumetric relaxation time (IVRT), Tei index (TEI), propagation velocity (Vp) and grade of mitral regurgitation on a scale 1–4 (MR). LVMI was calculated by dividing left ventricular mass (LVM) by body surface area (BSA).

### 2.5. Statistical Analysis

Statistical analysis was performed using Statistica 13.0 (StatSoft, Kraków, Poland). The Spearman rank correlation coefficient (Rs) was used for univariate analysis of the associations between the quantitative variables. Multiple linear regression was used for multivariate analysis after logarithmic transformation of the adipokine concentration values. The standardised β regression coefficients were calculated to compare the relative effect of each independent variable on the prognosis of the values of dependent variables. Comparisons of plasma adipokine concentrations of the CAD patients without and with HF were performed using the Mann–Whitney U test. A *p*-value < 0.05 was considered statistically significant.

## 3. Results

### 3.1. Correlations of Plasma Adipokine Concentrations to Clinical and Echocardiographic Parameters in Patients with CAD

The plasma concentrations of adiponectin, leptin and resistin were 5.25 ± 3.22 µg/mL, 15.3 ± 17.9 ng/mL and 7.81 ± 3.28 ng/mL, respectively (Table 1). The plasma adiponectin concentrations were significantly higher in patients with HF than in patients without HF, 6.08 ± 3.74 µg/mL vs. 4.65 ± 2.64 µg/mL. Leptin and resistin concentrations did not differ significantly between the groups (Table S1). We found several significant correlations of echocardiographic and clinical parameters with the analysed adipokine concentrations in the univariate analysis (Table 2). Correlations in the groups of CAD patients without and with HF are detailed in the Supplementary Materials (Table S2).

In the case of adiponectin concentrations, positive correlations were observed with age and severity of HF (as defined by the NYHA), as well as severity of CAD symptoms evaluated using the CCS, but also plasma BNP. Negative correlations with parameters of left ventricular systolic function, LVEF and LVSF, were observed. Positive correlations regarding the echocardiographic parameters were noted with MR, left ventricular end-diastolic diameter/body surface area (LVEDDBSA) and left ventricular end-diastolic volume/body surface area (LVEDVBSA).

Leptin concentration correlated positively with HF severity, as evaluated using NYHA, BMI, SBP and DBP; whereas, negative correlations were found with LVEDDBSA, Ao, IVRT and TEI.

In the case of resistin concentration, the only significant negative correlation was observed with E/A.

### 3.2. Associations of Echocardiographic Parameters in the Multivariate Regression Analysis

The multivariate analysis (Table 3, Table 4 and Table 5) revealed numerous significant associations between the echocardiographic parameters as dependent variables, and age, gender, T2DM, BMI, eGFR and logarithmically transformed adipokine concentrations as independent variables. We found that after adjustment for the mentioned confounders, LVEDV, LVEDD, LVESD and LAD were significantly positively associated with adiponectin concentration. In contrast, LVEF and LVSF were significantly negatively associated with adiponectin concentration. E/A was significantly positively associated with adiponectin and negatively with resistin concentration. None of the analysed echocardiographic parameters were associated with leptin concentration in the multivariate analysis. No associations were found between LVMI, IVSd, PWd, Ao, RVEDD, DT, IVRT, TEI, Vp and the analysed adipokine concentrations. Among the other independent variables that were significantly associated with the echocardiographic parameters in every model, gender, age and eGFR were the most significant variables. We showed that LVEDV, LVEDD and LVESD were significantly positively associated with male gender and negatively with eGFR. LAD was positively associated with male gender. LVEF and LVSF were significantly negatively associated with male gender, and LVEF was positively associated with eGFR. In the case of E/A, there was only negative association, which was with age. The loss of significance of all the associations of leptin concentration with the echocardiographic parameters in the multivariate analysis may be attributed to the role of gender, age and eGFR as confounders responsible for the correlations observed in the univariate analysis.

## 4. Discussion

Accumulating evidence corroborates that adipokines play an important role in the development and progression of CVD [5]. In our previous research, we documented the associations of plasma concentrations of interleukin 6 (IL-6) [39] and tumour necrosis factor α (TNF-α), together with its soluble receptors [38], with components of metabolic syndrome in patients with CAD. In the presented study, we hypothesised that there are associations between the plasma concentrations of the classic adipokines (adiponectin, leptin and resistin) with the parameters assessed in the echocardiography examinations of the same group of CAD patients. Indeed, we found multiple significant associations in both the univariate and multivariate analyses. The most noticeable are those regarding adiponectin and several parameters of cardiac function (LVEF, LVSF and E/A) and structure (LVEDD, LVESD, LVEDV and LAD), as well as resistin and E/A. It is also worth emphasising that although leptin was significantly positively correlated with HF severity (according to the NYHA), there were no significant associations with echocardiographic parameters in the multivariate model, including independent variables related to metabolic syndrome (T2DM, BMI) and renal function (eGFR).

CAD is one of the major risk factors for HF development. The main feature of HF and its extent is myocardial remodelling. Initially, an adaptive and functional mechanism allows the heart to maintain its function in harmful conditions, such as ischaemic injury and pressure or volume overload. However, a persistent response of myocardium cells, myocytes and interstitial cells to noxious haemodynamic, metabolic and inflammatory stimuli leads to alterations of energy metabolism and structure and contractile dysfunction, resulting in the progression of HF [41]. In addition to the classic mediators of cardiac remodelling, such as neurohormonal activation, inflammation and oxidative stress, adipokines form another class of suspected mediators. It is suggested that the imbalanced profile of adipokine secretion in patients with CAD is associated with the development and progression of HF [42,43,44]. However, information regarding their exact involvement in myocardial remodelling is still scarce.

### 4.1. Adiponectin

The associations of adiponectin with cardiac dysfunction and remodelling have been considered in many research studies. In patients without advanced CVD, but with obesity or HT, a decrease in adiponectin level is the cause of harmful changes in the heart [45,46,47,48]. Adiponectin secreted locally in small amounts by cardiomyocytes is particularly important for cardioprotection. Protective action is expressed through the limitations of cardiac lipotoxicity and myocardial oxidative stress, and the inhibition of pathological cardiac remodelling, with AMP-activated protein kinase (AMPK) as a central mediator of adiponectin signalling [4,49,50]. Piestrzeniewicz et al. [51] suggested that low adiponectin concentration can be considered a predictor of left ventricular remodelling in male patients one year after acute myocardial infarction (MI) successfully treated with primary percutaneous coronary intervention. In contrast, in advanced CVD, it has not been established whether an increase in adiponectin level is a compensatory mechanism that protects the cardiovascular system, a response to adiponectin resistance or a component of HF pathomechanism [11,12,13]. In our study, almost half of the patients had confirmed HF and higher adiponectin concentrations than those without HF (6.08 ± 3.74 µg/mL vs. 4.65 ± 2.64 µg/mL, respectively). Adiponectin concentrations showed positive correlations with age, severity of HF and plasma BNP concentrations. These correlations have been described previously [11,12,13]. An explanation is that BNP is a strong stimulus for the secretion of adiponectin from adipose tissue in patients with CAD [20] and HF [52]. Other conditions that are associated with increased adiponectin concentrations that may coexist with HF include cachexia and impaired renal function [13].

We also found that the positive correlation of plasma adiponectin concentration with severity of HF is followed by independent associations with parameters of systolic and diastolic function and remodelling features. Among them were inverse relationships of plasma adiponectin concentration with LVEF and LVSF, as well as positive associations with E/A, LVEDD, LVESD, LVEDV and LAD. The relationships between adiponectin level and left ventricular systolic and diastolic dysfunction have also been studied by other authors. The inverse association of adiponectin level with LVEF was indicated in elderly men [53] or with LVSF in patients with hypertrophic cardiomyopathy [54], but other studies in patients with HF did not confirm this [55,56,57]. Additional evidence confirms that adiponectin levels are positively correlated with indicators of diastolic dysfunction: left ventricular pressure half-time in patients with hypertrophic cardiomyopathy [58], E/E’ ratio in HF patients with metabolic syndrome [55] or CVD patients [57] and DT in CAD patients [59]. In the presented study, the positive association of adiponectin levels with E/A, but also the borderline negative associations with DT and IVRT, argue for a link to diastolic dysfunction with restrictive filling. According to our results, high adiponectin levels are independently associated with both left ventricular systolic and diastolic dysfunction. Furthermore, we have shown associations between adiponectin concentrations and echocardiographic features of left ventricular and left atrial remodelling, but reports on these issues are scarce. On one hand, positive correlations between adiponectin concentrations and LVEDD or LVESD have been found in patients with chronic HF and cardiac cachexia [60]. On the other hand, Farcaş et al. [43] questioned the associations between adiponectin concentration and the echocardiographic parameters of left ventricular remodelling in patients with CAD. Negative associations with interventricular septum thickness, left ventricular posterior wall thickness and left ventricular relative wall thickness, and a positive association with LVEDD lost their significance when the authors eliminated patients with DM from the analysis. It is noteworthy that in this study group, there was a greater percentage of women (50%) and the patients did not have histories of MI or HF, unlike many of the patients in our study group. This may explain the differences and emphasise the importance of past MI or HF. Moreover, according to our study, male gender and low eGFR are independent factors associated with left ventricular enlargement and systolic dysfunction. In contrast, the positive association of adiponectin concentrations with LAD in our study is consistent with other authors, in that they found positive relationships of adiponectin levels with LAD [22] and left atrial volume index (LAVI) [55,57] in HF patients. Increased left atrium size is an echocardiographic marker of atrial remodelling [61]. While the mechanisms by which adiponectin and CAD interact with each other remain elusive, multiple associations with echocardiographic parameters that are significant after adjustment for clinical factors suggest that adiponectin is a good candidate as a marker to improve the monitoring of cardiac remodelling and dysfunction in CAD patients.

### 4.2. Leptin

Leptin is another potential mediator of cardiac remodelling and HF. Our study indicated a positive correlation of plasma leptin levels with the severity of HF (as assessed by NYHA), but not with plasma BNP concentration. The lack of correlation between leptin levels and the levels of heart-failure-specific biomarkers, N-terminal pro-brain natriuretic peptide (NT-proBNP), mid-regional pro-atrial natriuretic peptide (MR-proANP) and galectin-3 was also found by Dadarlat et al. [62], who studied overweight and obese patients diagnosed with HF. These authors questioned the role of leptin as an independent predictor of HF prognosis in this group of patients.

Many researchers believe that leptin is involved in the pathogenesis of heart dysfunction in obese people because of its pro-inflammatory and pro-fibrotic effects, which impair cardiac metabolism and the functions of the myocardium and vasculature [10,31,63]. The pro-fibrotic effect of leptin results from increased collagen synthesis by cardiac fibroblasts with its reduced degradation [63]. Leptin levels are positively associated with reduced cardiac contractility [64], increased myocardial wall thickness [65] and left ventricular hypertrophy [66]. Moreover, in our study, leptin was the only one among the evaluated adipokines whose concentrations positively correlated with SBP and DBP. This is consistent with the notion that leptin is a mediator in obesity-induced HT via neurogenic mechanisms and the activation of the renin-angiotensin system, and thus may indirectly promote cardiac hypertrophy, fibrosis and ventricular remodelling [44,67].

In contrast, we found negative correlations between leptin concentrations and parameters such as LVEDD from BSA formula, IVRT and TEI, suggesting a beneficial effect of leptin, rather than a harmful one. However, in our multivariate analysis, we have not confirmed any independent associations between leptin concentration and echocardiographic parameters of cardiac remodelling or function. The position of other researchers on this issue is ambiguous. On one hand, the beneficial impact of leptin on the structure and function of the heart (left ventricular mass and volume, LAD or LVEF) has been suggested in multiethnic participants without CVD [68,69] and non-obese patients with CVD undergoing cardiovascular surgery [57]. The protective effect of leptin is also supported by a better prognosis (lower frequency of another cardiovascular event) among overweight or mildly obese patients with plasma leptin levels >2000 pg/mL [70]. On the other hand, there are reports that plasma leptin concentrations in CAD patients have been associated with echocardiographic parameters of ventricular remodelling (negative association with left ventricular posterior wall thickness and positive association with left ventricular relative wall thickness) [43] and impaired left ventricle diastolic (positive association with E/E’ and negative association with E/A), but not systolic, function [42,59]. High leptin levels predict the occurrence of major adverse cardiac events in patients with established CAD, independently of obesity and other cardiovascular risk factors [71]. These incompatibilities may be explained, in part, by the presence of confounding factors—obesity and related conditions, such as leptin resistance [72] and components of metabolic syndrome [34]. Obesity itself predisposes the individual to changes in cardiac morphology and ventricular function, which may lead to the development of HF with left ventricular hypertrophy and diastolic dysfunction [73]. Moreover, an important question is whether plasma leptin levels correspond to tissue effects. It is also unclear whether high leptin levels result in harmful cardiovascular effects or are a response to leptin resistance. Our study does not support the potential value of plasma leptin as a tool for monitoring of cardiac remodelling and function in CAD patients since it was not an independent marker for any of the studied echocardiographic parameters.

### 4.3. Resistin

Resistin has been proposed as a new potential contributing factor in the development and progression of HF. High levels of resistin are associated with an increased risk of new-onset HF [74,75,76], a more advanced NYHA degree of HF [77] and higher rates of mortality [78]. It has been assumed that resistin participates in the development of myocardium dysfunction via the promotion of insulin resistance and inflammation, but one study exists that suggests a different mechanism, independent of these factors [74]. In contrast, Farcas et al. [43] questioned the involvement of resistin in cardiac remodelling in patients with CAD without history of MI. In our study, we found a significant, but weak, negative correlation between resistin concentration and left ventricular diastolic function expressed by E/A. High resistin level is an independent factor associated with low E/A. This indicates that, unlike adiponectin, a high resistin level is associated with diastolic dysfunction with delayed left ventricular relaxation. The negative association of resistin level with E/A was also demonstrated in patients with T2DM [79] and CAD [59]. However, in a study with a large community sample with prevalent obesity, independent associations between resistin concentration and the parameters of left ventricular diastolic function were not found, while the authors suggested a role of resistin in the promotion of HF by the induction of systolic dysfunction [80]. Likewise, resistin levels were inversely related to LVFS in another community sample [81]. The discussed research is in connection with reports indicating that resistin directly affects the myocardium and mediates a decrease in both cardiomyocyte contractility and the velocities of contraction and relaxation, and promotes cardiac hypertrophy; however, these mechanisms of resistin action were mainly described in animal models [35,36,37,82]. Our results suggest that high plasma resistin might be a potential marker of diastolic function impairment; however, it has a rather weak association with E/A value. Ambiguous results of previous reports question its diagnostic value for cardiac remodelling and function in CAD patients.

### 4.4. Limitations

Our study has some limitations. First, as a cross-sectional study, it does not identify cause-effect relationships between adipokine levels and the echocardiographic parameters of cardiac structure and function. Secondly, it should be emphasised that our results were obtained from CAD patients with a high prevalence of positive history of MI and HF, so these results cannot be generalised to patients with less severe forms of CAD. Thirdly, all patients received optimal pharmacological treatment. The use of drugs, such as statins, might have affected plasma adipokine concentrations and metabolic parameters, although the results of the meta-analyses indicated the impact of statin therapy only on the plasma concentration of adiponectin [83], but not on leptin [84] and resistin [85].

## 5. Conclusions

In conclusion, the plasma concentrations of classical adipokines are associated with multiple echocardiographic parameters of cardiac structure and function in patients with CAD, suggesting that they might be involved in the mechanism of cardiac remodelling or, on the contrary, be part of a protective response to cardiac remodelling. Among these adipokines, adiponectin is of particular interest because its high concentrations were independently associated with features of systolic and diastolic dysfunction, as well as with structural indicators of cardiac remodelling. However, it is unclear whether adiponectin protects or participates in remodelling. Resistin concentrations were only negatively associated with E/A, which suggests a detrimental effect of resistin on left ventricular diastolic function. In addition, leptin concentrations were positively correlated with HF severity (as assessed by NYHA), but we found no evidence of its independent association with features of cardiac dysfunction and remodelling. The exact relationship between adipokines and the mechanism of heart remodelling in CAD patients remains unclear. Further studies on larger groups of patients are required to confirm our suggested potential benefits when it comes to using measurements of plasma concentrations of adiponectin and resistin as biochemical markers of cardiac dysfunction and remodelling in the monitoring of patients with CAD.

## Figures and Tables

**Table 1:** Characteristics of the study group (*n* = 167).

Parameter	Abbreviation	Value
Gender		133 M/34 F (80%/20%)
Age (years)		59.2 ± 8.4
Number of main coronary arteries with lesions		2.3 ± 1.0
Past myocardial infarction	MI	129 (77%)
Heart failure	HF	70 (42%)
Hypertension	HT	64 (38%)
Type 2 diabetes	T2DM	31 (19%)
Current smoking		24 (14%)
Asthma or chronic obstructive pulmonary disease		11 (7%)
Metabolic syndrome (ATP III)	MS ATP III	71 (43%)
Metabolic syndrome (IDF)	MS IDF	91 (54%)
Statin therapy		153 (92%)
Waist (cm)		96.5 ± 11.3
Waist-to-hip ratio	WHR	0.96 ± 0.08
Body mass index (kg/m^2^)	BMI	28.1 ± 4.1
Heart rate (beats/min)	HR	63.2 ± 6.9
Systolic blood pressure (mmHg)	SBP	126.8 ± 17.5
Diastolic blood pressure (mmHg)	DBP	79.0 ± 10.0
Blood haemoglobin (mmol/L)	HGB	8.61 ± 0.69
Fasting plasma glucose (mg/dL)	Glu	114.1 ± 32.4
Serum total cholesterol (mg/dL)	TCH	190.8 ± 38.5
Serum HDL cholesterol (mg/dL)	HDL	53.3 ± 15.2
Serum LDL cholesterol (mg/dL)	LDL	103.0 ± 29.5
Serum triacylglycerols (mg/dL)	TG	127.1 ± 81.7
Serum creatinine (mg/dL)	Crea	1.10 ± 0.18
Estimated glomerular filtration rate (mL/min/1.73 m^2^)	eGFR	70.5 ± 12.7
Serum uric acid (mg/dL)	UA	5.01 ± 1.45
Plasma brain natriuretic peptide (pmol/L)	BNP	231 ± 359
Plasma adiponectin (µg/mL)	Adipo	5.25 ± 3.22
Plasma leptin (ng/mL)	Lep	15.3 ± 17.9
Plasma resistin (ng/mL)	Res	7.81 ± 3.28
Left ventricular mass index (g/m^2^)	LVMI	148.0 ± 39.0
Left ventricular end-diastolic volume (ml)	LVEDV	142.0 ± 64.0
Left ventricular end-diastolic volume/BSA (mL/m^2^)	LVEDVBSA	72.8 ±31.8
Left ventricular end-diastolic diameter (mm)	LVEDD	57.7 ± 9.8
Left ventricular end-diastolic diameter/BSA (cm/m^2^)	LVEDDBSA	29.8 ± 5.1
Left ventricular end-systolic diameter (mm)	LVESD	43.0 ± 2.5
Intraventricular septal end-diastolic thickness (mm)	IVSd	10.5 ± 2.6
Posterior wall end-diastolic thickness (mm)	PWd	9.37 ± 1.89
Ascending aorta diameter (mm)	Ao	35.4 ± 3.7
Left atrium diameter (mm)	LAD	41.0 ± 6.5
Right ventricular end-diastolic diameter (mm)	RVEDD	21.9 ± 5.7
Right ventricular systolic pressure (mmHg)	RVSP	32.2 ± 9.8
Left ventricular ejection fraction (%)	LVEF	47.2 ± 16.1
Left ventricular shortening fraction (%)	LVSF	26.8 ± 10.3
Mitral valve E/A ratio	E/A	1.27 ± 0.85
Mitral valve deceleration time (ms)	DT	215 ± 91
Isovolumetric relaxation time (ms)	IVRT	104 ± 26
Tei index	TEI	0.64 ± 0.17
Propagation velocity (cm/s)	Vp	48.0 ± 17.5
Grade of mitral regurgitation	MR	1.47 ± 1.00

Data are presented as mean ± SD or number (percent). Abbreviations: MS ATP III—ATP III Diagnostic Criteria for Metabolic Syndrome, BSA—body surface area, MS IDF—International Diabetes Federation Consensus Worldwide Definition of the Metabolic Syndrome.

**Table 2:** Correlations of plasma adipokine concentrations with clinical and echocardiographic parameters in patients with CAD (Spearman rank correlation coefficients).

Correlated Parameters	Adiponectin		Leptin		Resistin	
	Rs	*p*-Value	Rs	*p*-Value	Rs	*p*-Value
Age	**0.36**	**0.0000018**	0.12	0.12	0.06	0.47
NYHA	**0.22**	**0.0039**	**0.19**	**0.016**	0.09	0.26
CCS	**0.17**	**0.028**	0.04	0.58	0.07	0.39
Number of MI	0.03	0.67	0.05	0.56	0.14	0.074
BMI	−0.08	0.30	**0.51**	**<0.000001**	0.02	0.83
HR	0.13	0.091	0.13	0.085	−0.02	0.80
SBP	0.15	0.052	**0.20**	**0.010**	0.00	0.96
DBP	0.08	0.31	**0.22**	**0.0037**	−0.01	0.86
BNP	**0.39**	**0.0000002**	−0.01	0.84	0.01	0.88
LVMI	0.06	0.47	−0.03	0.67	0.13	0.10
LVEDV	0.07	0.37	−0.08	0.28	0.06	0.47
LVEDVBSA	**0.15**	**0.049**	−0.14	0.07	0.07	0.35
LVEDD	0.09	0.26	−0.08	0.29	0.02	0.83
LVEDDBSA	**0.26**	**0.00057**	**−0.17**	**0.026**	0.05	0.54
LVESD	0.14	0.078	−0.04	0.65	0.06	0.48
IVSd	−0.10	0.19	0.08	0.31	0.14	0.063
PWd	−0.08	0.32	0.04	0.61	0.01	0.92
Ao	−0.13	0.087	**−0.16**	**0.042**	−0.05	0.50
LAD	0.13	0.089	0.08	0.29	0.01	0.89
RVEDD	0.02	0.79	0.08	0.33	0.06	0.42
RVSP	0.23	0.089	−0.02	0.90	0.05	0.69
LVEF	**−0.20**	**0.0093**	0.02	0.78	−0.06	0.45
LVSF	**−0.17**	**0.025**	−0.01	0.89	−0.09	0.27
E/A	0.11	0.18	−0.06	0.46	**−0.17**	**0.034**
DT	−0.14	0.072	−0.03	0.75	−0.04	0.64
IVRT	−0.15	0.057	**−0.21**	**0.0080**	−0.02	0.80
TEI	−0.03	0.72	**−0.20**	**0.014**	0.09	0.28
Vp	−0.14	0.083	0.09	0.28	−0.03	0.75
MR	**0.32**	**0.000035**	0.01	0.91	−0.03	0.72

Significant correlations (*p* < 0.05) are marked in bold. Abbreviations: Ao—ascending aorta diameter; BNP—plasma brain natriuretic peptide; CCS—severity of CAD symptoms evaluated by the Canadian Cardiovascular Society Angina Grading Scale; DBP—diastolic blood pressure; DT—mitral valve deceleration time; E/A—mitral valve E/A ratio; HR—heart rate beats/min; IVRT—isovolumetric relaxation time; IVSd—intraventricular septal end-diastolic thickness; LAD—left atrium diameter; LVEDD—left ventricular end-diastolic diameter; LVEDDBSA—left ventricular end-diastolic diameter/body surface area; LVEDV—left ventricular end-diastolic volume; LVEDVBSA—left ventricular end-diastolic volume/body surface area; LVEF—left ventricular ejection fraction; LVESD—left ventricular end-systolic diameter; LVMI—left ventricular mass index; LVSF—left ventricular shortening fraction; MI—myocardial infarction; MR—grade of mitral regurgitation, NYHA—severity of heart failure evaluated by the New York Heart Association functional classification; PWd—posterior wall end-diastolic thickness; RVEDD—right ventricular end-diastolic diameter; RVSP—right ventricular systolic pressure; SBP—systolic blood pressure; TEI—Tei index; Vp—propagation velocity.

**Table 3:** Multivariate linear regression models for echocardiography parameters as dependent variables and male gender, age, T2DM, BMI, eGFR and logarithm of plasma adiponectin concentration as independent variables.

Dependent Variables	Independent Variables											
	Male Gender		Age		T2DM		BMI		eGFR		Log Adiponectin	
	β (95% CI)	*p*-Value	β (95% CI)	*p*-Value	β (95% CI)	*p*-Value	β (95% CI)	*p*-Value	β (95% CI)	*p*-Value	β (95% CI)	*p*-Value
LVMI	**+0.25** **(+0.08–+0.43)**	**0.0044**	+0.05 (−0.13–+0.23)	0.58	+0.10 (−0.06–+0.27)	0.23	+0.15 (−0.01–+0.31)	0.061	−0.07 (−0.25–+0.12)	0.48	+0.16 (−0.01–0.34)	0.069
LVEDV	**+0.50** **(+0.34–+0.66)**	**<0.000001**	−0.12 (−0.28–+0.04)	0.15	+0.09 (−0.07–+0.24)	0.26	+0.03 (−0.11–+0.18)	0.65	**−0.31** **(−0.48–−0.15)**	**0.00029**	**+0.29** **(+0.13–+0.45)**	**0.00037**
LVEDD	**+0.46** **(+0.29–+0.62)**	**<0.000001**	−0.06 (−0.23–+0.11)	0.48	+0.09 (−0.06–+0.25)	0.25	+0.07 (−0.08–+0.22)	0.37	**−0.20** **(−0.37–−0.03)**	**0.024**	**+0.26** **(0.10–+0.43)**	**0.0020**
LVESD	**+0.45** **(+0.29–+0.62)**	**<0.000001**	−0.07 (−0.24–+0.09)	0.39	+0.15 (−0.01–+0.30)	0.061	+0.05 (−0.10–+0.20)	0.50	**−0.21** **(−0.38–−0.03)**	**0.019**	**+0.30** **(+0.14–+0.46)**	**0.00030**
IVSd	+0.06 (−0.12–+0.23)	0.51	+0.12 (−0.05–+0.30)	0.16	+0.04 (−0.12–+0.21)	0.60	**+0.24** **(+0.07–+0.40)**	**0.0044**	+0.04 (−0.14–+0.22)	0.65	−0.11 (−0.28–+0.07)	0.22
PWd	−0.09 (−0.26–+0.09)	0.34	+0.06 (−0.12–+0.24)	0.50	+0.02 (−0.15–+0.19)	0.79	**+0.22** **(+0.06–+0.38)**	**0.0082**	+0.08 (−0.11–+0.26)	0.42	−0.07 (−0.25–+0.10)	0.41
Ao	**+0.55** **(+0.39–+0.71)**	**<0.000001**	**+0.17** **(+0.01–+0.32)**	**0.040**	+0.01 (−0.14–+0.16)	0.87	+0.02 (−0.12–+0.17)	0.75	**−0.20** **(−0.37–−0.04)**	**0.017**	−0.05 (−0.20–+0.11)	0.55
LAD	**+0.31** **(+0.14–+0.49)**	**0.00039**	−0.01 (−0.18–+0.16)	0.89	+0.07 (−0.09–+0.24)	0.38	+0.16 (0.00–+0.31)	0.054	−0.14 (−0.32–+0.04)	0.11	**+0.21** **(+0.04–+0.38)**	**0.015**
RVEDD	**+0.28** **(+0.11–+0.46)**	**0.0016**	+0.01 (−0.17–+0.19)	0.89	−0.05 (−0.21–+0.12)	0.56	**+0.19** **(+0.03–+0.35)**	**0.020**	−0.08 (−0.27–+0.10)	0.36	+0.12 (−0.06–+0.29)	0.18
LVEF	**−0.41** **(−0.57–−0.24)**	**0.000002**	+0.05 (−0.12–+0.21)	0.56	**−0.17** **(−0.33–−0.02)**	**0.031**	+0.01 (−0.14–+0.16)	0.91	**+0.21** **(+0.04–+0.38)**	**0.017**	**−0.32** **(−0.49–−0.16)**	**0.00012**
LVSF	**−0.39** **(−0.56–−0.23)**	**0.000005**	+0.09 (−0.08–+0.26)	0.28	**−0.17** **(−0.32–−0.01)**	**0.035**	−0.04 (−0.19–+0.11)	0.63	+0.17 (0.00–+0.34)	0.055	**−0.31** **(−0.48–−0.15)**	**0.00027**
E/A	+0.17 (−0.01–+0.35)	0.062	**−0.35** **(−0.52–−0.18)**	**0.00009**	+0.07 (−0.09–0.24)	0.38	+0.03 (−0.13–+0.19)	0.71	−0.03 (−0.21–+0.15)	0.71	**+0.40** **(+0.23–+0.57)**	**0.000008**
DT	+0.07 (−0.10–+0.25)	0.40	**+0.32** **(+0.15–+0.50)**	**0.00031**	**+0.21** **(+0.05–+0.38)**	**0.011**	−0.08 (−0.24–+0.08)	0.34	+0.04 (−0.14–+0.22)	0.64	−0.16 (−0.33–+0.02)	0.074
IVRT	**+0.19** **(+0.01–+0.37)**	**0.039**	**+0.24** **(+0.06–+0.42)**	**0.0088**	+0.04 (−0.13–+0.20)	0.67	−0.10 (−0.26–+0.07)	0.25	−0.08 (−0.26–+0.11)	0.40	−0.17 (−0.35–0.00)	0.056
TEI	**+0.33** **(+0.15–+0.51)**	**0.00051**	−0.03 (−0.20–+0.15)	0.78	+0.11 (−0.06–+0.28)	0.20	−0.15 (−0.32–+0.01)	0.061	**−0.29** **(−0.48–−0.10)**	**0.0026**	+0.01 (−0.16–+0.19)	0.89
Vp	**−0.32** **(−0.49–−0.14)**	**0.00036**	−0.17 (−0.34–0.00)	0.051	**−0.21** **(−0.38–−0.05)**	**0.0099**	**+0.16** **(0.00–+0.32)**	**0.046**	**+0.21** **(+0.04–+0.39)**	**0.018**	−0.16 (−0.33–+0.01)	0.071

Significant associations (*p* < 0.05) are marked in bold. Abbreviations: Ao—ascending aorta diameter; BMI—body mass index; DT—mitral valve deceleration time; E/A—mitral valve E/A ratio; eGFR—estimated glomerular filtration rate; IVRT—isovolumetric relaxation time; IVSd—intraventricular septal end-diastolic thickness; LAD—left atrium diameter; LG ADIPO—logarithmically transformed adiponectin concentrations; LVEDD—left ventricular end-diastolic diameter; LVEDV—left ventricular end-diastolic volume; LVEF—left ventricular ejection fraction; LVESD—left ventricular end-systolic diameter; LVMI—left ventricular mass index; PWd—posterior wall end-diastolic thickness; RVEDD—right ventricular end-diastolic diameter; LVSF—left ventricular shortening fraction; T2DM—type 2 diabetes mellitus; TEI—Tei index; Vp—propagation velocity; β—standardised beta coefficient in multiple linear regression model.

**Table 4:** Multivariate linear regression models for echocardiography parameters as dependent variables and male gender, age, T2DM, BMI, eGFR and logarithm of plasma leptin concentration as independent variables.

Dependent Variables	Independent Variables											
	Male Gender		Age		T2DM		BMI		eGFR		Log Leptin	
	β (95% CI)	*p*-Value	β (95% CI)	*p*-Value	β (95% CI)	*p*-Value	β (95% CI)	*p*-Value	β (95% CI)	*p*-Value	β (95% CI)	*p*-Value
LVMI	+0.13 (−0.08–+0.34)	0.23	+0.08 (−0.09–+0.26)	0.33	+0.08 (−0.08–+0.25)	0.32	**+0.24** **(+0.03–+0.45)**	**0.022**	−0.12 (−0.31–+0.07)	0.20	−0.19 (−0.44–+0.06)	0.14
LVEDV	**+0.50** **(+0.30–+0.69)**	**0.000002**	−0.03 (−0.20–+0.13)	0.67	+0.02 (−0.13–+0.18)	0.75	−0.04 (−0.24–+0.15)	0.66	**−0.33** **(−0.50–−0.15)**	**0.00036**	+0.13 (−0.11–+0.37)	0.27
LVEDD	**+0.42** **(+0.22–+0.62)**	**0.00005**	+0.01 (−0.16–+0.17)	0.91	+0.04 (−0.12–+0.20)	0.61	+0.03 (−0.17–+0.22)	0.80	**−0.23** **(−0.41–−0.04)**	**0.015**	+0.06 (−0.18–+0.30)	0.61
LVESD	**+0.44** **(+0.24–+0.64)**	**0.00003**	+0.01 (−0.15–+0.18)	0.90	+0.09 (−0.07–+0.25)	0.26	−0.03 (−0.23–+0.17)	0.76	**−0.23** **(−0.41–−0.04)**	**0.015**	+0.13 (−0.11–+0.37)	0.29
IVSd	+0.05 (−0.16–+0.25)	0.65	+0.09 (−0.08–+0.26)	0.28	+0.07 (−0.10–+0.23)	0.41	**+0.28** **(+0.08–+0.49)**	**0.0076**	+0.04 (−0.14–+0.23)	0.65	−0.08 (−0.33–+0.17)	0.53
PWd	−0.15 (−0.36–+0.06)	0.15	+0.04 (−0.14–+0.21)	0.68	+0.05 (−0.12–+0.21)	0.57	**+0.32** **(+0.11–+0.52)**	**0.0031**	+0.06 (−0.13–+0.24)	0.56	−0.18 (−0.43–+0.08)	0.17
Ao	**+0.61** **(+0.43–+0.80)**	**<0.000001**	**+0.16** **(0.00–+0.31)**	**0.044**	+0.01 (−0.13–+0.16)	0.85	−0.03 (−0.21–+0.16)	0.75	**−0.18** **(−0.34–−0.01)**	**0.040**	+0.11 (−0.11–+0.33)	0.34
LAD	**+0.34** **(+0.13–+0.54)**	**0.0014**	+0.05 (−0.12–+0.22)	0.57	+0.02 (−0.14–+0.19)	0.77	+0.06 (−0.14–+0.27)	0.54	−0.15 (−0.33–+0.04)	0.12	+0.16 (−0.09–+0.41)	0.21
RVEDD	**+0.35** **(+0.14–+0.56)**	**0.0011**	+0.05 (−0.12–+0.22)	0.55	−0.08 (−0.24–+0.08)	0.34	+0.08 (−0.12–+0.29)	0.42	−0.07 (−0.25–+0.12)	0.49	+0.20 (−0.06–+0.45)	0.13
LVEF	**−0.38** **(−0.58–−0.18)**	**0.00027**	−0.04 (−0.20–+0.13)	0.65	−0.10 (−0.26–+0.06)	0.20	+0.08 (−0.12–+0.28)	0.42	**+0.24** **(+0.05–+0.42)**	**0.011**	−0.11 (−0.35–+0.13)	0.37
LVSF	**−0.40** **(−0.61–−0.20)**	**0.00015**	+0.00 (−0.16–+0.17)	0.97	−0.11 (−0.27–+0.05)	0.19	+0.07 (−0.14–+0.27)	0.52	+0.18 (0.00–+0.37)	0.05	−0.17 (−0.41–+0.08)	0.18
E/A	+0.09 (−0.14–+0.32)	0.44	**−0.25** **(−0.43–−0.08)**	**0.0054**	−0.01 (−0.18–+0.16)	0.91	−0.02 (−0.25–+0.20)	0.83	−0.06 (−0.25–+0.14)	0.55	+0.07 (−0.20–+0.34)	0.61
DT	+0.11 (−0.10–+0.32)	0.30	**+0.28** **(+0.12–+0.45)**	**0.0011**	**+0.25** **(+0.08–+0.41)**	**0.0030**	−0.06 (−0.27–+0.15)	0.58	+0.06 (−0.13–+0.24)	0.55	−0.02 (−0.28–+0.24)	0.88
IVRT	+0.15 (−0.06–+0.37)	0.17	**+0.20** **(+0.02–+0.37)**	**0.027**	+0.08 (−0.09–+0.24)	0.36	+0.01 (−0.20–+0.23)	0.91	−0.09 (−0.28–+0.10)	0.35	−0.19 (−0.45–+0.07)	0.16
TEI	**+0.34** **(+0.12–+0.57)**	**0.0027**	−0.02 (−0.19–+0.15)	0.81	+0.11 (−0.06–+0.27)	0.20	−0.17 (−0.38–+0.04)	0.11	**−0.29** **(−0.47–−0.10)**	**0.0033**	+0.03 (−0.23–+0.29)	0.81
Vp	**−0.25** **(−0.45–−0.04)**	**0.017**	**−0.21** **(−0.37–−0.04)**	**0.015**	**−0.18** **(−0.34–−0.02)**	**0.028**	+0.14 (−0.06–+0.34)	0.18	**+0.24** **(+0.05–+0.42)**	**0.011**	+0.05 (−0.20–+0.29)	0.71

Significant associations (*p* < 0.05) are marked in bold. Abbreviations: Ao—ascending aorta diameter; BMI—body mass index; DT—mitral valve deceleration time; E/A—mitral valve E/A ratio; eGFR—estimated glomerular filtration rate; IVRT—isovolumetric relaxation time; IVSd—intraventricular septal end-diastolic thickness; LAD—left atrium diameter; LG LEP—logarithmically transformed leptin concentrations; LVEDD—left ventricular end-diastolic diameter; LVEDV—left ventricular end-diastolic volume; LVEF—left ventricular ejection fraction; LVESD—left ventricular end-systolic diameter; LVMI—left ventricular mass index; PWd—posterior wall end-diastolic thickness; RVEDD—right ventricular end-diastolic diameter; LVSF—left ventricular shortening fraction; T2DM—type 2 diabetes mellitus; TEI—Tei index; Vp—propagation velocity; β—standardised beta coefficient in multiple linear regression model.

**Table 5:** Multivariate linear regression models for echocardiography parameters as dependent variables and male gender, age, T2DM, BMI, eGFR and logarithm of plasma resistin concentration as independent variables.

Dependent Variables	Independent Variables											
	Male Gender		Age		T2DM		BMI		eGFR		Log Resistin	
	β (95% CI)	*p*-Value	β (95% CI)	*p*-Value	β (95% CI)	*p*-Value	β (95% CI)	*p*-Value	β (95% CI)	*p*-Value	β (95% CI)	*p*-Value
LVMI	**+0.20** **(+0.03–+0.37)**	**0.019**	+0.11 (−0.06–+0.28)	0.22	+0.07 (−0.09–+0.23)	0.40	+0.14 (−0.02–+0.30)	0.09	−0.03 (−0.22–+0.17)	0.77	+0.14 (−0.02–+0.30)	0.085
LVEDV	**+0.42** **(+0.26–+0.59)**	**<0.000001**	−0.03 (−0.19–+0.13)	0.70	+0.03 (−0.12–+0.18)	0.70	+0.02 (−0.13–+0.17)	0.77	**−0.32** **(−0.50–−0.14)**	**0.00072**	+0.07 (−0.08–+0.23)	0.35
LVEDD	**+0.39** **(+0.23–+0.56)**	**0.000006**	+0.01 (−0.16–+0.17)	0.94	+0.04 (−0.11–+0.20)	0.58	+0.06 (−0.10–+0.21)	0.47	**−0.24** **(−0.43–−0.05)**	**0.014**	0.00 (−0.16–+0.15)	0.97
LVESD	**+0.38** **(+0.21–+0.54)**	**0.00001**	+0.01 (−0.16–+0.17)	0.91	+0.10 (−0.06–+0.25)	0.24	+0.04 (−0.12–+0.19)	0.66	**−0.24** **(−0.42–−0.05)**	**0.014**	+0.03 (−0.13–+0.19)	0.71
IVSd	+0.073 (−0.10–+0.24)	0.39	+0.11 (−0.06–+0.28)	0.18	+0.06 (−0.11–+0.22)	0.49	**+0.24** **(+0.08–+0.40)**	**0.0043**	+0.12 (−0.07–+0.31)	0.22	+0.15 (−0.01–+0.31)	0.070
PWd	−0.07 (−0.24–+0.11)	0.45	+0.04 (−0.13–+0.21)	0.64	+0.04 (−0.13–+0.20)	0.66	**+0.23** **(+0.06–+0.39)**	**0.0071**	+0.08 (−0.12–+0.28)	0.42	−0.01 (−0.18–+0.15)	0.86
Ao	**+0.57** **(+0.41–+0.72)**	**<0.000001**	+0.15 (−0.01–+0.30)	0.063	+0.02 (−0.12–+0.17)	0.74	+0.03 (−0.12–+0.17)	0.70	**−0.22** **(−0.40–−0.05)**	**0.013**	−0.06 (−0.21–+0.08)	0.38
LAD	**+0.27** **(+0.10–+0.44)**	**0.0023**	+0.04 (−0.13–+0.21)	0.67	+0.04 (−0.13–+0.20)	0.66	**+0.15** **(−0.01–+0.31)**	**0.07**	**−0.20** **(−0.39–0.00)**	**0.047**	−0.05 (−0.21–+0.11)	0.54
RVEDD	**+0.25** **(+0.08–+0.42)**	**0.0048**	+0.05 (−0.12–+0.23)	0.54	−0.07 (−0.23–+0.09)	0.39	**+0.18** **(+0.02–+0.34)**	**0.027**	−0.06 (−0.26–+0.13)	0.52	+0.09 (−0.07–+0.25)	0.27
LVEF	**−0.33** **(−0.49–−0.16)**	**0.00015**	−0.04 (−0.20–+0.13)	0.68	−0.11 (−0.27–+0.05)	0.17	+0.03 (−0.13–+0.18)	0.75	**+0.25** **(+0.06–+0.44)**	**0.010**	−0.01 (−0.17–+0.14)	0.87
LVSF	**−0.32** **(−0.49–−0.15)**	**0.00025**	+0.01 (−0.16–+0.18)	0.94	−0.11 (−0.27–+0.05)	0.16	−0.02 (−0.18–+0.14)	0.81	**+0.20** **(+0.01–+0.39)**	**0.041**	−0.03 (−0.19–+0.13)	0.68
E/A	+0.05 (−0.12–+0.23)	0.55	**−0.27** **(−0.45–−0.10)**	**0.0023**	−0.01 (−0.17–+0.16)	0.94	+0.02 (−0.15–+0.19)	0.81	−0.14 (−0.34–+0.06)	0.17	**−0.19** **(−0.36–−0.03)**	**0.023**
DT	+0.12 (−0.05–+0.29)	0.17	**+0.28** **(+0.11–+0.45)**	**0.0013**	**+0.25** **(+0.08–+0.41)**	**0.0031**	−0.07 (−0.23–+0.09)	0.40	+0.05 (−0.15–+0.24)	0.62	−0.03 (−0.19–+0.14)	0.75
IVRT	**+0.24** **(+0.06–+0.42)**	**0.0077**	**+0.20** **(+0.03–+0.38)**	**0.025**	+0.07 (−0.09–+0.24)	0.39	−0.09 (−0.26–+0.08)	0.29	−0.04 (−0.24–+0.16)	0.67	+0.04 (−0.12–+0.21)	0.62
TEI	**+0.33** **(+0.15–+0.51)**	**0.00039**	−0.02 (−0.19–+0.15)	0.84	+0.11 (−0.06–+0.27)	0.20	**−0.16** **(−0.32–+0.01)**	**0.06**	**−0.28** **(−0.47–−0.08)**	**0.005**	+0.03 (−0.13–+0.19)	0.69
Vp	**−0.27** **(−0.44–−0.11)**	**0.0015**	**−0.21** **(−0.37–−0.04)**	**0.016**	**−0.18** **(−0.34–−0.02)**	**0.030**	**+0.16** **(0.00–+0.32)**	**0.048**	**+0.24** **(+0.05–+0.43)**	**0.014**	+0.02 (−0.14–+0.18)	0.77

Significant associations (*p* < 0.05) are marked in bold. Abbreviations: Ao—ascending aorta diameter; BMI—body mass index; DT—mitral valve deceleration time; E/A—mitral valve E/A ratio; eGFR—estimated glomerular filtration rate; IVRT—isovolumetric relaxation time; IVSd—intraventricular septal end-diastolic thickness; LAD—left atrium diameter; LG RES—logarithmically transformed resistin concentrations; LVEDD—left ventricular end-diastolic diameter; LVEDV—left ventricular end-diastolic volume; LVEF—left ventricular ejection fraction; LVESD—left ventricular end-systolic diameter; LVMI—left ventricular mass index; PWd—posterior wall end-diastolic thickness; RVEDD—right ventricular end-diastolic diameter; LVSF—left ventricular shortening fraction; T2DM—type 2 diabetes mellitus; TEI—Tei index; Vp—propagation velocity; β—standardised beta coefficient in multiple linear regression model.

## Data Availability

The data presented in this study are available on request from the corresponding author.

## Supplementary Materials

The following are available online at https://www.mdpi.com/article/10.3390/diagnostics11101774/s1, Table S1: Comparisons of adipokine concentrations of CAD patients without and with HF. Table S2: Correlations between plasma adipokine concentrations and echocardiographic parameters in CAD patients without or with HF.
