# Supplementary material for: Association of Adiponectin, Leptin and Resistin Plasma Concentrations with Echocardiographic Parameters in Patients with Coronary Artery Disease
Source: Diagnostics (Basel). 2021 Sep 26;11(10):1774. doi: 10.3390/diagnostics11101774 (PMC8534895; doi:10.3390/diagnostics11101774)
Supplement: Supplementary file 1 [file diagnostics-11-01774-s001.zip › diagnostics-1402211-supplementary.pdf]

Table S1. Comparisons of adipokine concentrations of CAD patients without and with HF.

Adipokine	Patients without HF (n=97)	Patients with HF (n=70)	*p*-Value
Adiponectin [µg/mL]	4.65 ± 2.64	6.08 ± 3.74	**0.004731**
Leptin [ng/mL]	15.41 ± 19.93	15.07 ± 14.85	0.586948
Resistin [ng/mL]	7.80 ± 3.19	7.83 ± 3.42	0.811578

Data are presented as mean ± SD
Significant differences (p<0.05) are marked in bold.

Table S2. Correlations between plasma adipokine concentrations and echocardiographic parameters in CAD patients without or with HF.

Parameter	Adiponectin				Leptin				Resistin			
	Correlations for Patients without HF (n=97)		Correlations for Patients with HF (n=70)		Correlations for Patients without HF (n=97)		Correlations for Patients with HF (n=70)		Correlations for Patients without HF (n=97)		Correlations for Patients with HF (n=70)	
	Rs	p-value	Rs	p-value	Rs	p-value	Rs	p-value	Rs	p-value	Rs	p-value
LVMI	-0.08	0.454	0.04	0.735	0.05	0.605	-0.19	0.118	0.06	0.549	**0.31**	**0.010**
LVEDV	-0.18	0.083	0.13	0.298	**-0.23**	**0.025**	-0.03	0.780	-0.13	0.216	**0.41**	**0.000489**
LVEDVBSA	-0.03	0.791	0.20	0.094	**-0.30**	**0.00277**	-0.13	0.297	-0.09	0.387	**0.46**	**0.000092**
LVEDD	-0.08	0.465	0.11	0.383	**-0.20**	**0.047**	-0.02	0.855	-0.16	0.122	**0.24**	**0.049**
LVEDDBSA	**0.20**	**0.049**	0.22	0.0717	**-0.26**	**0.00879**	-0.20	0.094	-0.06	0.549	0.20	0.094
LVESD	-0.05	0.632	0.10	0.400	-0.16	0.120	-0.01	0.933	-0.03	0.782	0.24	0.047
IVSd	-0.03	0.784	-0.12	0.305	**0.25**	**0.014**	-0.12	0.340	0.17	0.095	0.14	0.255
PWd	-0.13	0.199	0.07	0.593	0.11	0.285	0.01	0.938	0.07	0.509	-0.06	0.613
Ao	**-0.25**	**0.013**	0.005	0.970	**-0.23**	**0.022**	-0.07	0.567	**-0.21**	**0.040**	0.15	0.230
LAD	-0.02	0.850	0.19	0.110	0.07	0.483	0.06	0.650	-0.02	0.820	0.08	0.517
RVEDD	-0.10	0.327	0.16	0.191	0.08	0.409	0.09	0.475	-0.01	0.959	0.14	0.251
RVSP	0.05	0.809	0.21	0.226	0.28	0.181	-0.07	0.700	0.04	0.851	0.10	0.591
LVEF	-0.08	0.447	-0.12	0.313	0.11	0.297	0.04	0.730	0.06	0.579	**-0.36**	**0.002116**
LVSF	0.04	0.698	-0.15	0.212	0.06	0.552	0.03	0.831	-0.06	0.574	**-0.25**	**0.039**
E/A	-0.11	0.301	**0.32**	**0.013**	-0.17	0.092	0.07	0.573	**-0.23**	**0.022**	-0.13	0.332
DT	-0.08	0.416	-0.19	0.140	0.13	0.193	-0.22	0.082	-0.01	0.931	-0.06	0.643
IVRT	-0.06	0.573	**-0.28**	**0.026**	-0.16	0.129	**-0.28**	**0.023**	-0.06	0.588	0.06	0.629
TEI	-0.13	0.214	0.02	0.910	-0.18	0.083	**-0.28**	**0.032**	-0.03	0.799	**0.38**	**0.003**
Vp	-0.10	0.320	0.13	0.321	0.13	0.224	0.22	0.085	-0.03	0.799	-0.15	0.253
MR	0.16	0.126	**0.43**	**0.00021**	-0.03	0.748	0.02	0.875	-0.20	0.057	0.23	0.057

Significant correlations (p<0.05) are marked in bold.
Abbreviations: Ao – ascending aorta diameter; DT – mitral valve deceleration time; E/A – mitral valve E/A ratio; IVRT – isovolumetric relaxation time; IVSd – intraventricular septal end-diastolic thickness; LAD – left atrium diameter; LVEDD – left ventricular end-diastolic diameter; LVEDDBSA – left ventricular end-diastolic diameter/body surface area; LVEDV – left ventricular end-diastolic volume; LVEDVBSA – left ventricular end-diastolic volume/body surface area; LVEF – left ventricular ejection fraction; LVESD – left ventricular end-systolic diameter; LVMI – left ventricular mass index; LVSF – left ventricular shortening fraction; MR – grade of mitral regurgitation, PWd – posterior wall end-diastolic thickness; RVEDD – right ventricular end-diastolic diameter; RVSP – right ventricular systolic pressure; TEI – Tei index; Vp – propagation velocity.
